# Supplementary material for: WordCommentsAnalyzer: A windows software tool for qualitative research
Source: F1000Res. 2018 Sep 4;7:536. Originally published 2018 May 3. [Version 2] doi: 10.12688/f1000research.14819.2 (PMC6137411; doi:10.12688/f1000research.14819.2)
Supplement: Supplementary file 3 [file f1000research-7-17614-s0002.tgz › b2a79496-4ada-47c4-9682-a99ce867836d.zip!/Eight Iranian Health Journals/Salmand_Iranian Journal of Ageing _32.docx]

© Salmand_Iranian Journal of Ageing . This work is licensed under CC BY-NC 4.0 (https://creativecommons.org/licenses/by-nc/4.0/).

Volume & Issue: دوره 5 - شماره 3

Title: «سلامت عمومی» زنان مراقبت کننده از سالمند مبتلا به آلزایمر

http://salmandj.uswr.ac.ir/article-1-276-fa.html

اهداف: مطالعه حاضر با هدف بررسی سلامت عمومی زنان مراقبت کننده از سالمند مبتلا به آلزایمر انجام شد. مواد و روش‌ها: این مطالعه توصیفی–مقطعی، بخش مقدماتی از یک مطالعه کارآزمایی در عرصه بود که حجم نمونه آن 150 نفر برآورد شد و افراد مورد نظر، به روش نمونه گیری مستمر انتخاب شدند. جامعه پژوهش در این مطالعه شامل زنانی بود که بین 18 تا 60 سال سن داشته، از وابستگان درجه یک سالمند مبتلا به آلزایمر بوده و مراقبت مستقیم از سالمند را به مدت حداقل 6 ماه به عهده داشتند. بررسی سلامت عمومی در پژوهش، با پرسش نامه 28 آیتمی سلامت عمومی (GHQ-28) انجام شد. تجزیه و تحلیل داده ها، با آزمون های t، خی دو، کروس کالوالیس و MANOVA انجام شد. یافته‌ها: نتایج نشان داد که %24 از زنان مورد مطالعه دارای سلامت عمومی مطلوب بوده و 58/7% سلامت عمومی متوسط و 17/3% دارای سلامت عمومی نامطلوب بودند. بین متغیر سلامت عمومی زنان مراقبت کننده و مشخصات سن (P=0/016)، وضعیت اشتغال (P=0/008)، نسبت خویشاوندی (P=0/013) و وضعیت اقتصادی (P=0/049) ارتباط معناداری وجود داشت. اما بین سلامت عمومی و عواملی همچون وضعیت تاهل (P=0/5)، سطح تحصیلات (P=0/07)، و مدت مراقبت از سالمند مبتلا به آلزایمر (P=0/205)  ارتباط معنادار یافت نشد. نتیجه‌گیری: پژوهش حاضر نشان داد که درصد قابل توجهی از زنان مراقبت کننده از سالمند مبتلا به آلزایمر در وضعیت مطلوبی از سلامت عمومی قرار نداشتند. لذا با توجه به اینکه در ایران، مطالعات جنسیت محور در مراقبین کمتر صورت گرفته، پیشنهاد می شود تحقیقات بعدی بر ابعاد مختلف سلامتی و ارتقا آن در مردان و زنان مراقب انجام گیرد.

Title: اثر 6 هفته برنامه تمرینی ثبات مرکزی بر میزان زمین خوردن سالمندان

http://salmandj.uswr.ac.ir/article-1-274-fa.html

اهداف: هدف این پژوهش بررسی 6 هفته برنامه تمرینی ثبات مرکزی بر میزان زمین خوردن سالمندان است. مواد و روش‌ها: در این کارآزمایی بالینی، 40 نفر از زنان سالمند (میانگین سن: 66/20±5/98 سال، وزن: 62/60±5/78 کیلوگرم، قد: 159/94±7/8 سانتی متر)، به طور داوطلبانه در تحقیق شرکت کردند. آزمودنی ها یک هفته قبل از شروع تمرینات ثبات مرکزی، بر اساس نمرات آزمون تعادلی برگ به دو گروه «با خطر زمین خوردن کم» (گروه 1) و «با خطر زمین خوردن زیاد» (گروه 2) تقسیم شدند. سپس به صورت تصادفی به دو گروه تجربی و کنترل تقسیم شدند. گروه های تجربی، تمرینات ثبات مرکزی (شامل سه سطح) را انجام دادند. مدت تمرین در هر جلسه تمرین، حدود 30 دقیقه و طول دوره تمرینی 6 هفته (به صورت یک روز در میان) بود. پس از اتمام تمرینات، پس آزمون تعادل برگ در همان محیط از گروه های کنترل  تجربی به عمل آمد. به منظور تحلیل داده ها از آزمون های آماری t مستقل و t وابسته در سطح معنی دار 0/05 استفاده شد. یافته‌ها: نتایج نشان داد که بین گروه تجربی و کنترل در گروه های با خطر زمین خوردن کم و زیاد (گروه های 1 و 2) در پیش آزمون اختلاف معنی داری وجود نداشت (P=0/486 و P=0/193) اما در پس آزمون، گروه های تجربی عملکرد بهتری نسبت به گروه های کنترل داشتند (P=0/007 و P=0/001). میزان پیشرفت در گروه تجربی با خطر زمین خوردن کم و زیاد به ترتیب برابر %3/38 و %15/06 و برای گروه های کنترل به ترتیب %0/95 و %3/7 بود. نتیجه‌گیری: یافته های تحقیق نشان دادند که تمرینات ثبات مرکزی می تواند کنترل پاسچر و در نتیجه میزان زمین خوردن را در سالمندان بهبود بخشد.

Title: مقایسه سلامت عمومی سالمندان مقیم یا غیر مقیم سرای سالمندان

http://salmandj.uswr.ac.ir/article-1-273-fa.html

اهداف: تحقیق حاضر با هدف مقایسه سلامت عمومی و شاخصه های آن در سالمندان مقیم و غیر مقیم سرای سالمندان انجام شد. مواد و روش‌ها: مطالعه از نوع مقطعی مقایسه ای و شرکت کنندگان در پژوهش 254 سالمند ) 127 نفر مقیم سرای سالمندان و 127 نفر مقیم جامعه) بودند. شرکت کنندگان به شیوه نمونه گیری در دسترس انتخاب شدند و در هر گروه 83 زن و 44 مرد به تناسب سهم جنسیت در جامعه قرار گرفتند. مقیاس سلامت عمومی (GHQ-28) که در برگیرنده سلامت عمومی و چهار خرده مقیاس اضطراب، افسردگی، نشانه‌های جسمانی، و کارکرد اجتماعی است برای جمع آوری اطلاعات مورد استفاده قرار گرفت. از آزمون هایt  و تحلیل واریانس برای تحلیل نتایج استفاده شد. یافته‌ها: نتایج نشان داد میانگین نمرات سلامت عمومی سالمندان مقیم سرای سالمندان از میانگین نمرات سالمندان مقیم جامعه بیشتر (نمره بالا نشانه سلامت روان پایین) بود و تفاوت مشاهده شده در میانگین ها برای سلامت عمومی و زیر مقیاس های نشانه های افسردگی و جسمانی و اختلال در کارکرد اجتماعی از لحاظ آماری معنادار به دست آمد. اختلال در کارکرد اجتماعی زنان مقیم آسایشگاه بیشتر از مردان بود. نتیجه‌گیری: یافته های پژوهش، حاکی از آن است که سالمندان مقیم سرای سالمندان در مقایسه با سالمندان مقیم جامعه نشانه های افسردگی، جسمانی و اختلالات کارکرد اجتماعی بیشتری را تجربه می کنند. از سویی گسترش موسسات نگهداری سالمندی در آینده غیر قابل اجتناب است بنابراین، توجه هر چه بیشتر به شرایط محیط زندگی سالمندان مقیم موسسات ضروری به نظر می رسد.

Title: تأثیر خاطره گویی بر سلامت روانی سالمندان

http://salmandj.uswr.ac.ir/article-1-280-fa.html

اهداف: هدف از مطالعه حاضر، بررسی تاثیر خاطره گویی بر سلامت روانی سالمندان بود. مواد و روش‌ها: پژوهش حاضر مطالعه ای نیمه تجربی بود که 57 نفر از سالمندان مقیم آسایشگاه سالمندان شهید هاشمی نژاد تهران با نمونه گیری تصادفی طبقه ای شرکت کردند. معیارهای ورود به مطالعه سن 60 سال و بالاتر، کسب نمره سلامت روان بین 0 تا 66/6 )سلامت روان ضعیف و متوسط)، تکلم به زبان فارسی، توانایی شنیداری و گفتاری قابل قبول، جهت شرکت در جلسات، زندگی تمام وقت در آسایشگاه، نداشتن سابقه بستری شدن در بیمارستان روانی، درمان روانی و تجربه سوگ، طی 6 ماه گذشته، تمایل به شرکت در تحقیق، هوشیاری نسبت به زمان، مکان و شخص، اقامت در آسایشگاه حداقل به مدت شش ماه و عدم دریافت هر گونه درمانی که در توانایی ذهنی، حافظه یا تفکر اختلال ایجاد کند. سطح سلامت روان شرکت کنندگان قبل از مداخله، به وسیله پرسشنامه 28 سوالی سلامت عمومی گلدبرگ ارزیابی شد. مداخله شامل هشت جلسه خاطره گویی، هفته ای دوبار (به مدت 1-1/5 ساعت) بود. در این جلسات سالمندان به بیان خاطرات خوشایند گذشته پرداختند، سپس سطح سلامت روان شرکت کنندگان، مجددا اندازه گیری شد. داده ها با استفاده از نرم افزار SPSS نسخه 16 مورد تجزیه و تحلیل قرار گرفت و از آزمون t زوجی، جهت مقایسه داده ها استفاده شد. یافته‌ها: یافته های مطالعه نشان داد بین نمرات قبل و بعد از مداخله در تمامی ابعاد سلامت روان (نشانه های جسمانی، اضطراب و اختلالات خواب، عملکرد اجتماعی و نشانه های افسردگی) و همچنین نمره کل سلامت روان اختلاف معنی داری (P=0/001)  وجود داشت. نتیجه‌گیری: خاطره گویی بر بهبود سلامت روان سالمندان مقیم آسایشگاه موثر است؛ لذا می توان از این تکنیک ساده، قابل اجرا و کم هزینه در آسایشگاه های سالمندان استفاده کرد.

Title: ساختار عاملی آزمون ارزیابی عملکردی راه رفتن در سالمندان ایرانی

http://salmandj.uswr.ac.ir/article-1-272-fa.html

اهداف: پژوهش حاضر با هدف بررسی ساختار عاملی آزمون ارزیابی عملکردی راه رفتن در سالمندان ایرانی انجام شد. مواد و روش‌ها: پژوهش علّی‌مقایسه ای حاضر با 200 آزمودنی سالمند در دامنه سنی 60 سال به بالا در دو گروه با و بدون سابقه زمین خوردن (هر گروه، 100 آزمودنی) انجام شد که به صورت داوطلبانه انتخاب شدند. معیار ورود به مطالعه کسب حداقل نمره 24 از 30 در مقیاس ارزیابی حداقل میزان هشیاری(MMSE)  بود. شرکت کنندگان آیتم های موجود در آزمون ارزیابی عملکردی راه رفتن را انجام داده، اطلاعات توسط یک محقق جمع آوری شد. آزمون ارزیابی عملکردی راه رفتن از 10 آیتم تشکیل شده است که وضعیت عملکردی فرد را در یک دامنه 0 تا 30  به روش لیکرت اندازه گیری می کند. داده ها با استفاده از تحلیل عامل اکتشافی و تحلیل مولفه های اصلی با چرخش واریماکس و نیز آزمون t مستقل ارزیابی شدند. یافته‌ها: نتایج تحقیق نشان داد که آزمون ارزیابی عملکردی راه رفتن از دو عامل تشکیل شده است. این دو عامل در کل52/53 درصد واریانس را پیش بینی می کردند که بر اساس رسم نمودار ارزش های ویژه (نمودار اسکری) و الگوی وزن های عاملی پیشنهاد شدند. تمامی سوال ها بار عاملی بالاتر از حد پذیرش(0/5)  داشتند به طوری که دامنه این مقادیر در کل آزمون در دامنه 0/523  تا 0/846 متغیر بود. نتیجه‌گیری: نتایج تحقیق نشان داد که نسخه فارسی آزمون ارزیابی عملکردی راه رفتن در سالمندان ایرانی نیز در حد قابل قبولی هماهنگ با نسخه انگلیسی آن بوده، می توان از آن برای شناسایی سالمندان در خطر زمین خوردن استفاده کرد.

Title: تأثیر 4 هفته برنامه بازتوانی بر افزایش دامنه حرکتی و کاهش درد در مردان مبتلا به شانه منجمد

http://salmandj.uswr.ac.ir/article-1-268-fa.html

اهداف: هدف از این مطالعه، تعیین اثربخشی برنامه های بازتوانی حرکت درمانی و الکتروتراپی بر افزایش دامنه حرکتی و کاهش درد شانه منجمد مردان مبتلا به نوع ایدیوپاتیک بود. مواد و روش‌ها: تحقیق حاضر از نوع نیمه تجربی بود و نمونه شامل 18 بیمار (با میانگین سنی 56/4±6/7 سال، قد 175/1±5/3 سانتی متر، وزن 73/1±7/5 کیلوگرم و سابقه بیماری 9/2±5/1 ماه) مراجعه کننده به کلینیک های شهر ایلام بود. معیارهای ورود به مطالعه شامل: جنسیت مذکر، ابتلا به بیماری نوع ایدیوپاتیک شانه منجمد، از دست رفتن قابل توجه تحرک، وجود درد شبانه و طی فعالیت در مفصل شانه بود. معیارهای خروج از مطالعه شامل: ابتلا به بیماری شانه منجمد ثانویه، داشتن سابقه بیماری آسیب های زمینه ای موثر بر شانه منجمد و سابقه استفاده از هر نوع تدابیر درمانی جهت رفع مشکلات شانه منجمد بود. از پرسشنامه اطلاعات شخصی، مقیاس VAS جهت ارزیابی میزان درد و گونیامتر برای اندازه گیری دامنه حرکتی مفصل شانه در پژوهش استفاده شد. برنامه بازتوانی روزانه به مدت 1 ساعت به صورت منظم در طی 4 هفته، شامل برنامه های الکتروتراپی (50 دقیقه) و حرکت درمانی (60 دقیقه) به صورت روز در میان اجرا شد. تجزیه و تحلیل داده ها با آزمون t زوجی انجام شد. یافته‌ها: نتایج نشان داد که میزان درد شبانه و طی فعالیت، بعد از 4 هفته انجام تمرینات بازتوانی، کاهش معناداری داشت (P<0/05). اما افراد مبتلا همچنان با میزانی از درد در زمان فعالیت و استراحت روبرو بودند و به طور کامل درد آنها تسکین نیافت. نتایج همچنین بیانگر آن بود که دامنه حرکتی مفصل شانه در حرکات خم شدن، دور شدن، چرخش خارجی و داخلی در پس آزمون بهبود معناداری یافت .(P<0/05) هرچند که افراد مبتلا همچنان با محدودیت حرکتی در مفصل شانه مواجه بودند. دامنه حرکتی شانه در پس آزمون نسبت به پیش آزمون در حرکات خم شدن 49/4±21/2  درجه، دور شدن 42/8±15/6  درجه، چرخش خارجی 28/3±10/4  درجه و چرخش داخلی 22/6±10/6  درجه بهبود نشان داد. نتیجه‌گیری: انجام تمرینات بازتوانی الکتروتراپی و حرکت درمانی به طور معناداری بر کاهش درد و بهبود دامنه حرکتی مفصل شانه بیماران در حرکات خم شدن، دور شدن، چرخش داخلی و خارجی اثر دارد. بنابراین می توان از برنامه های بازتوانی جهت کاهش درد و افزایش دامنه حرکتی شانه بیماران مبتلا به شانه منجمد بهره برد.

Title: مشکلات ارتباطی سالمندان مبتلا به آلزایمر و مراقبت‌کنندگان اصلی

http://salmandj.uswr.ac.ir/article-1-275-fa.html

اهداف: این مطالعه با هدف تعیین مشکلات ارتباطی سالمندان مبتلا به آلزایمر و مراقبت کنندگان اصلی آنها صورت گرفت. مواد و روش‌ها: مطالعه حاضر پژوهشی توصیفی است که 100 مراقبت کننده اصلی دارای ملاک های پژوهش به روش سرشماری از انجمن آلزایمر تهران انتخاب شدند. ابزار جمع آوری داده ها، پرسشنامه پژوهشگر ساخته حاوی 47 سوال بود و منعکس کننده مشکلات ارتباطی مراقبت کننده (فهمیدن، درک سخنان، درخواست ها، نیازها، معنا و مفهوم اعمال و رفتار بیمار، اشکال در فهماندن درخواست ها و موضوعات به بیمار) بود. جهت بررسی روایی پرسشنامه از روش اعتبار محتوا و به منظور بررسی پایایی از دو روش بازآزمایی (r=%95) و تعیین همسانی درونی آلفای کرونباخ 0/96 استفاده شد. همچنین شدت اختلال ذهنی سالمند مبتلا به آلزایمر با استفاده از ابزار استاندارد شده ارزیابی وضعیت روانشناختی فرم کوتاه تعیین شد. تجزیه و تحلیل داده ها با استفاده از نرم افزار SPSS و آزمون t انجام شد. یافته‌ها 74% :مراقبت کنندگان، مشکل خود در برقراری ارتباط با بیمار را در حد بالا،17%  درحد متوسط و 9%  درحد کم بیان کردند .73% از افراد مورد پژوهش بیشترین میزان مشکلات ارتباطی را در حیطه فهماندن درخواست ها و موضوعات به بیمار و %27 بیشترین میزان مشکل را حیطه فهمیدن و درک درخواست ها، نیازها، معنا و مفهوم اعمال و رفتار بیمار گزارش کردند. بین سن و جنس مراقبت کنندگان و نیز شدت اختلال ذهنی سالمند مبتلا به آلزایمر با مشکلات ارتباطی موجود ارتباط معنی داری وجود داشت، به طوری که با افزایش سن مراقبت کننده، جنسیت مونث مراقبت و افزایش شدت بیماری سالمند، شدت مشکلات ارتباطی افزایش نشان داد .(P<0/05) نتیجه‌گیری: از آنجا که برقراری ارتباط موثر، عاملی مهم در برآورده کردن نیازهای جسمی و روحی است و با توجه به نتایج پژوهش حاضر که حاکی از بالا بودن وجود مشکلات ارتباطی مراقبین و بیماران بود، ضروریست افرادی که از سالمندان مبتلا به آلزایمر مراقبت می کنند در زمینه راهکارها و مهارت های ارتباطی ویژه، جهت برقراری ارتباط با سالمند مبتلا به آلزایمر، آموزش دیده تا بتوانند به بهترین نحو از آنها مراقبت کرده، خود نیز دچار خستگی و فرسودگی ناشی از مراقبت از این بیماران نشوند.

Title: وضعیت سلامت سالمندان تحت پوشش کمیته امداد امام خمینی (ره)

http://salmandj.uswr.ac.ir/article-1-279-fa.html

اهداف: هدف از مطالعه حاضر بررسی وضعیت سلامت سالمندان تحت پوشش کمیته امداد امام خمینی بود. مواد و روش‌‌ها: مطالعه حاضر از نوع توصیفی تحلیلی بود نمونه شامل 250 سالمند تحت پوشش کمیته امداد امام خمینی (ره) شهرستان بیرجند بود که با روش نمونه گیری غیر احتمالی آسان و به تناسب سهمی از بین مراجعه کنندگان به نواحی 1 و 2 کمیته امداد شهر بیرجند انجام شد. از پرسشنامه طرح جامع سلامت سالمندان استفاده شد. پرسشنامه از طریق مصاحبه با سالمندان تکمیل و قد، وزن، فشارخون در شرایط استاندارد اندازه گیری شد. داده ها با نرم افزار Spss و آزمون آماری کای اسکوئر در سطح 0/05 مورد بررسی قرار گرفت. یافته‌ها: نتایج نشان داد که میانگین سنی سالمندان شرکت کننده71±8/7 سال 47/6%) زن و%52/4  مرد) بود. سالمندان بی سواد و بقیه با سواد بودند، 41/6% سالمندان مشکل مسکن، 11/2% مشکل بیمه،%93/2%  مشکل اقتصادی داشتند .(10/4%) سابقه ابتلا به دیابت و 39/6% مبتلا به پرفشاری خون بودند. 68% مشکل بینایی و 10/8% اختلال ادرار داشتند. 68/4% مبتلا به افسردگی خفیف و 3/2% دارای افسردگی شدید و 24/4% مبتلا به اختلالات شناختی شدید تا خفیف بودند. 24% سالمندان مبتلا به سوء تغذیه و 67/6%  ریسک ابتلا به سوء تغذیه را داشتند. شیوع پرفشاری خون در مردان بیشتر از زنان(P=0/07)  و در ساکنین شهری بیشتر از روستایی (P=0/04) بود. شیوع افسردگی در بین زنان بیشتر از مردان(P<0/001)  و در ساکنین نقاط شهری بیشتر از مناطق روستایی بود.(P=0/02)  از نظر وضعیت فعالیتهای روزانه نیز اختلاف معنی داری بین مردان و زنان مشاهده شد. نتیجه‌گیری: شیوع بالای مسایل اقتصادی و معیشتی سالمندان، می تواند سایر مشکلات جسمی و روحی آنان را دامن زده یا تشدید کند و لزوم توجه بیشتر مسوولین بهداشت و درمان، کمیته امداد و سایر ارگان ها را به قشر سالمند تحت پوشش برای جلوگیری از وخامت اوضاع پررنگ می کند.
